# Estimation of Handgrip Force from SEMG Based on Wavelet Scale Selection

**DOI:** 10.3390/s18020663

**Published:** 2018-02-24

**Authors:** Kai Wang, Xianmin Zhang, Jun Ota, Yanjiang Huang

**Affiliations:** 1 Guangdong Provincial Key Laboratory of Precision Equipment and Manufacturing Technology, South China University of Technology, Guangzhou 510640, China; 201410100131@mail.scut.edu.cn (K.W.); zhangxm@scut.edu.cn (X.Z.); 2 Research into Artifacts, Center for Engineering, University of Tokyo, Chiba 113-8654, Japan; ota@race.u-tokyo.ac.jp

**Keywords:** surface electromyography, handgrip force, force-varying muscle contraction, nonlinear analysis, wavelet scale selection

## Abstract

This paper proposes a nonlinear correlation-based wavelet scale selection technology to select the effective wavelet scales for the estimation of handgrip force from surface electromyograms (SEMG). The SEMG signal corresponding to gripping force was collected from extensor and flexor forearm muscles during the force-varying analysis task. We performed a computational sensitivity analysis on the initial nonlinear SEMG-handgrip force model. To explore the nonlinear correlation between ten wavelet scales and handgrip force, a large-scale iteration based on the Monte Carlo simulation was conducted. To choose a suitable combination of scales, we proposed a rule to combine wavelet scales based on the sensitivity of each scale and selected the appropriate combination of wavelet scales based on sequence combination analysis (SCA). The results of SCA indicated that the scale combination VI is suitable for estimating force from the extensors and the combination V is suitable for the flexors. The proposed method was compared to two former methods through prolonged static and force-varying contraction tasks. The experiment results showed that the root mean square errors derived by the proposed method for both static and force-varying contraction tasks were less than 20%. The accuracy and robustness of the handgrip force derived by the proposed method is better than that obtained by the former methods.

## 1. Introduction

Various dynamic handgrip tasks are required in a number of applications, including manual fabrication and machining, handling tools, monitoring for surgery, human–robot interactions and so on [1,2,3]. Usually, these tasks require long duration muscle contractions. With respect to the characteristics of usage, a high level of repetition and force is required in the handgrip tasks, which can be considered a risk factor for work-related upper extremity muscular disorder (WUED) [4]. Therefore, accurately monitoring the handling force in real time is important for preventing WUED [5,6]. To obtain real-time handgrip force measurements, one indirect measurement method is based on surface electromyography (SEMG), which is a common, non-invasive technique for analyzing muscle contractions for real-world applications [4,7,8]. However, SEMG signals are affected by physiological factors and non-physiological factors during prolonged muscle contractions, which affects force estimation from SEMG [1,9,10]. These influence factors are complex and varied under a prolonged muscle contraction environment. Therefore, it is necessary to obtain valid signals from the SEMG to realize accurate prediction of handgrip force in a long duration gripping motion.

In previous studies, a number of correlations between SEMG of a target muscle and force output of a target task have been discussed. In muscle contraction, the activation of motor units within a target muscle follows a mechanism (motor unit rotation and substitution) to maintain a given force. However, muscle fatigue resulting from prolonged muscle contractions can reduce the force generation performance of the target muscles. To overcome the decline in performance, more fresh motor units are recruited to replace the burden of fatigued motor units and compensate for a lack of performance [7,9,11,12]. Many researchers have reported that the increase in amplitude of SEMGs collected from long duration muscle contractions is caused by complex electrophysiological events (i.e., motor unit rotation or substitution), motion artifact, and so on, which, as noises, affect the SEMG signals at different frequencies and reduce the force estimation performance [8,13,14,15,16,17]. According to this phenomenon, many time-frequency and time-scale techniques have been used to eliminate the influence of these noises on force estimation from SEMG.

Many researchers used Fast Fourier Transform (FFT) to observe that the power spectrum of SEMG shifts to a lower frequency range when complex noises (electrophysiological effect of fatigue, random noises) develops [18,19,20,21]. The frequency banding technique based on FFT showed that the low frequency band amplitude is increased linearly during endurance isometric contraction tasks [22]. Kumar et al. (2003) found that the amplitude of the SEMG signal in the high frequency band does not change significantly with the development of complex physiological noise, through wavelet analysis [23]. To analyze the changes in SEMG in varying muscle contractions, time-frequency techniques have typically been adopted. A comparison of different time-frequency techniques indicated that continuous wavelet transformation (CWT) produces precise results with good representation of time and frequency localization [24]. The frequency-band analysis technique based on wavelet transformation has been generally used to explore the valid signals from SEMG for accurately estimating force. Sparto et al. (1999) used different wavelet scales of the Daubechies (db) wavelet function of order 6 to report that a decrease in high frequency wavelet coefficients and an increase in low frequency wavelet coefficients may be correlated with fatiguing muscle contractions [25]. Soo et al. (2010) performed a comparison of 55 types of wavelet scale combinations (WSC) and reported that a correlation between two scales (scale 2 and scale 3 of db2) and handgrip force provides a stable estimate of handgrip force from SEMG during endurance isometric contractions [6].

Many CWT-based methods, such as frequency band analysis, have been utilized to obtain a suitable wavelet scale for estimating accurate force from SEMG. These methods have used a single scale or particular WSC to analyze and filter the original signal [14,22]. An original signal can be decomposed into signals under different wavelet scales (i.e., different frequency band ranges) and retains the time domain features through CWT. In practical grasping tasks, each wavelet scale signal contains a variety of complex physiological and non-physiological factors. It is difficult to identify which factors are present at different scales. Currently, the nonlinear differences in quantification between each wavelet scale and the measured force are not clear. Thus, it is necessary to analyze the nonlinear correlations (i.e., nonlinear degree) between each wavelet scale and force in the estimation of handgrip force from SEMG.

The purpose of this paper is to find an appropriate WSC for accurate evaluation of handgrip force from SEMG during force-varying muscle contractions of long duration. The suitable WSC was obtained by using the sequence combination analysis (SCA) method based on Monte Carlo sensitivity analysis. As a simple and reliable sensitivity analysis method, Monte Carlo simulation can calculate the degree of nonlinearity between variables and the actual target output, regardless of how many factors the variable contains [26,27]. We first obtained the nonlinear correlations between wavelet scales and handgrip force during prolonged force-varying muscle contraction in the Monte Carlo sensitivity analysis, and then selected the appropriate WSC by analyzing the nonlinearity degree of each correlation. Finally, we evaluated the proposed method by comparing it to former methods. The root mean square error (RMSE) was used to evaluate the difference between the estimated force and the measured force during the WSC selection procedure, as well as comparisons between the proposed method and former methods. The contribution of this study is that we have investigated the nonlinear correlations between the wavelet scale and handgrip force. These correlations can enhance the selection of an appropriate WSC to accurately estimate handgrip force during force-varying muscle contractions of long duration.

## 2. Experimentation and Methodology

### 2.1. Experimental Setup

Twenty healthy participants (16 men and 4 women), whose ages ranged from 22 to 31 years, participated in the experiment. At this stage, we did not consider the differences between males and females. Ten subjects (group A: 8 men and 2 women) were used for determining the suitable WSC for estimating handgrip force in the force-varying contractions as a training group. The other ten subjects (group B: 8 men and 2 women) were used as a validation group, to evaluate the performance of the suitable WSC. Before the experiment, all participants were informed of the experiment process and the risk of muscle fatigue, and agreed to perform the experiment. 

**Figure 1:**
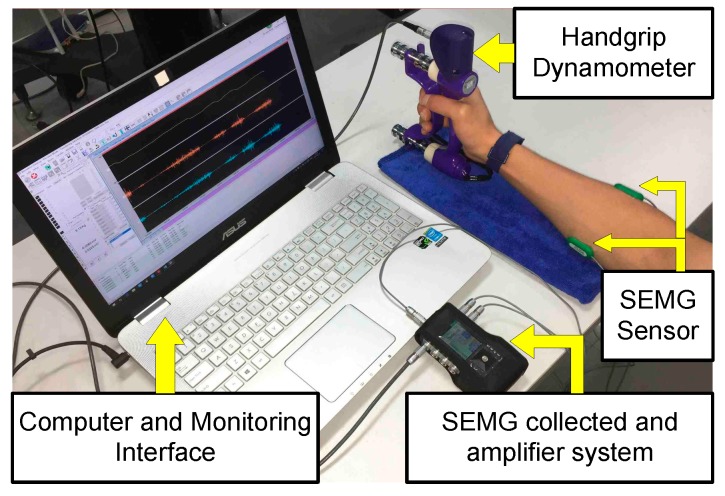
Experimental setup. SEMG: surface electromyogram.

In the gripping tasks, SEMG data was recorded from the flexor digitorum superficialis (FDS) muscle and extensor carpi radialis (ECR) muscle of the forearm, as shown in Figure 1. These are the dominant forearm muscles that contribute to handgrip force [28,29]. The SEMG was measured using reusable integral bipolar dry surface electrodes (Green Sensor Electrodes SX230, Biometrics Ltd., Newport, UK) with a skin contact area of 10 mm^2^ for each electrode and a center-to-center recording distance of 20 mm. Electrode locations were determined in accordance with a previous study [6,29]. EMG signals were amplified 20 times and bandpass prefiltered (15–450 Hz), together with the force signals that were obtained at a sampling rate of 1000 Hz (14 bits), using a portable electrophysiological amplifier system (Biometrics Ltd., DataLOG-MWX8-Bluetooth, UK, input resistance > 1015 ohms, CMRR-60 Hz > 96 dB) and stored on a computer. Handgrip forces (kg) were measured using an adjustable handgrip dynamometer. The sampling rate was set to 1000 Hz, and the data was digitized at a 14-bit resolution. To reduce the influence of grip width, a grip width of 65 mm was selected [5]. The force signal was visualized online, on a computer screen, to provide feedback about the exerted force by subjects during the data collection procedure, as shown in Figure 1.

### 2.2. Experimental Procedure

At the beginning of the experiment, each participant was asked to perform a maximal voluntary contraction task (MVC) three times, and from this, the average value of maximum grip strength (kg) and maximum amplitude (mV) of the SEMG were calculated. In the experiment, the participants in Group A first performed the force-varying analysis task to obtain the suitable WSC using the proposed method. Then, all participants in Groups A and B performed the static and force-varying validation tasks to evaluate the performance of the proposed method. The force-varying analysis task, static validation task, and force-varying validation task are defined as follows.

#### 2.2.1. Force-Varying Analysis Task (Force-Varying Contraction)

**Figure 2:**
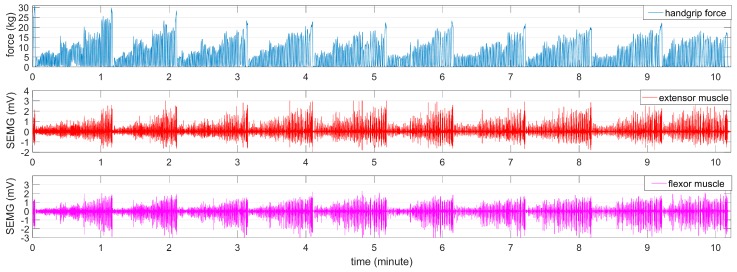
The process of the force-varying analysis task.

Participants in Group A were required to perform a force-varying fluctuating contraction. In the force-varying analysis task, a series of varying handgrip forces, occurring within ten minutes, was increased or decreased respectively by squeezing and releasing the dynamometer. Participants were asked to increase their handgrip force gradually to provide an adequate correlation between handgrip force and SEMG for each minute, as shown in Figure 2.

#### 2.2.2. Static Validation Task (Isometric Muscle Contraction)

Static tasks were performed at four force levels, which included 20%, 30%, 50%, and 70% of the MVC in a random order during the isometric contraction [6,29]. The participants sustained these contractions until the generated force dropped below 5% from the target force level for 5 s. The experiment with each force level was executed only once. To avoid the effect of muscle fatigue, participants were allowed to rest for 15 min before performing the next force level experiment [6].

#### 2.2.3. Force-Varying Validation Task (Voluntary Contraction)

**Figure 3:**
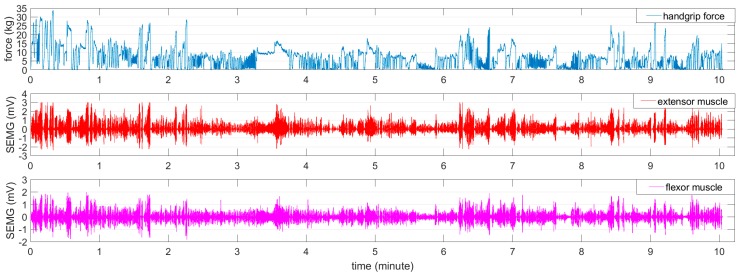
The process of the force-varying validation task.

In the force-varying validation task, a force level that varied between 0% and 80% of the MVC was performed over a 10-min period. Before the force-varying validation task, the participant rested for 60 min. A sample of the recorded handgrip force and SEMG for both muscles is illustrated in Figure 3.

### 2.3. Proposed Method

To accurately estimate the handgrip force from the SEMG signals during the long duration force-varying grip motion, this paper utilized the Monte Carlo sensitivity analysis to determine the nonlinear degree of the relationship between each wavelet scale and handgrip force from the initial nonlinear SEMG-handgrip force relationship, and used a nonlinear correlation-based scale-selection method (SCA) to obtain the suitable WSC of the scale.

#### 2.3.1. Initial Nonlinear SEMG-Handgrip Force Relationship

The initial nonlinear SEMG-handgrip force relationship was built using the ten wavelet (db2) scales (scale number: 1 2, 3, 4, 5, 6, 7, 8, 9, 10) from the SEMG signals and handgrip force collected from the force-varying analysis tasks of group A. These wavelet scales are most relevant to handgrip force [23]. This model is based on the second-order polynomial [29,30]. Given the predictors *e*(1), …, *e*(*P*), the response of handgrip force *F*(*estimation*) is predicted by:

(1) $$F\left(estimation\right)=\overset{P}{\underset{p=1}{\sum }}\left(\theta_{1p}e\left(p\right)+\theta_{2p}e^{2}\left(p\right)\right)\;$$

where 1≤p≤P=10, *P* is the total number of wavelet scales, and the vectors of coefficients $\theta_{1}=\left(\theta_{11},\ldots ,\theta_{1p},\ldots ,\theta_{1P}\right)$ and $\theta_{2}=\left(\theta_{21},\ldots ,\theta_{2p},\ldots ,\theta_{2P}\right)$ are produced by ordinary least squares. In this paper, the predictors, e(p), are the wavelet coefficients’ intensities of the wavelet scale of *p*, which are collected from the original SEMG *x_n_,* with data size, *N*, through CWT and root mean square (RMS)

(2) $$c_{n}\left(p\right)=\overset{N-1}{\underset{n=0}{\sum }}x_{n}\psi^{*}\left[\frac{\left(n^{\prime }-n\right)\delta t}{p}\right]$$

where 0≤n≤N−1, $c_{n}\left(p\right)$ is the wavelet coefficient, δt is the sampling time, ψ is the mother wavelet, and (^*^) indicates the complex conjugate [31]. Then, the valid intensity of wavelet coefficients can be calculated after pre-processing of the RMS with the window frame size of *M*

(3) $$e_{n}\left(p\right)=\sqrt{\frac{1}{M}\overset{\left(k+1\right)M-1}{\underset{n=kM}{\sum }}c_{n}{\left(p\right)}^{2}}$$

where $0\leq k\leq \frac{N}{M}-1$. A moving average filter (200 ms) [32] was used to smooth the $e_{n}\left(p\right)$.

#### 2.3.2. Sensitivity Analysis

For a nonlinear index, the sensitivity analysis is important [26]. In contrast to other sensitivity analysis methods, Monte Carlo simulation, as a variance-based global sensitivity analysis method, can obtain the sensitivity of interactions between the parameters [27]. This paper used the multivariate Monte Carlo simulation to obtain the sensitivity value of each wavelet scale related to force, predicted from the original nonlinear model (Equation (1)). The Monte Carlo simulation is often used to calculate the expected estimation of parameters in known mathematical models with random variables [26,27]. The main steps of Monte Carlo simulation can be categorized as follows:

(1) CWT was used to convert the original SEMG signals to wavelet coefficients of the ten scales in the force-varying analysis task.
(2) Wavelet coefficients of the ten scales normalized by MVC data were used to build the initial nonlinear SEMG-handgrip force relationship.
(3) A specific number of random variables were used to conduct a simulation of the handgrip force in the initial nonlinear relationship.
(4) Mean square deviation was used to calculate the simulated sensitivity value corresponding to each correlation between ten scales and handgrip force.
(5) The sensitivity value of each correlation was normalized by the sum of the sensitivity values of the ten scales.
(6) Steps 1–5 were repeated for every minute in the force-varying analysis task.

**Figure 4:**
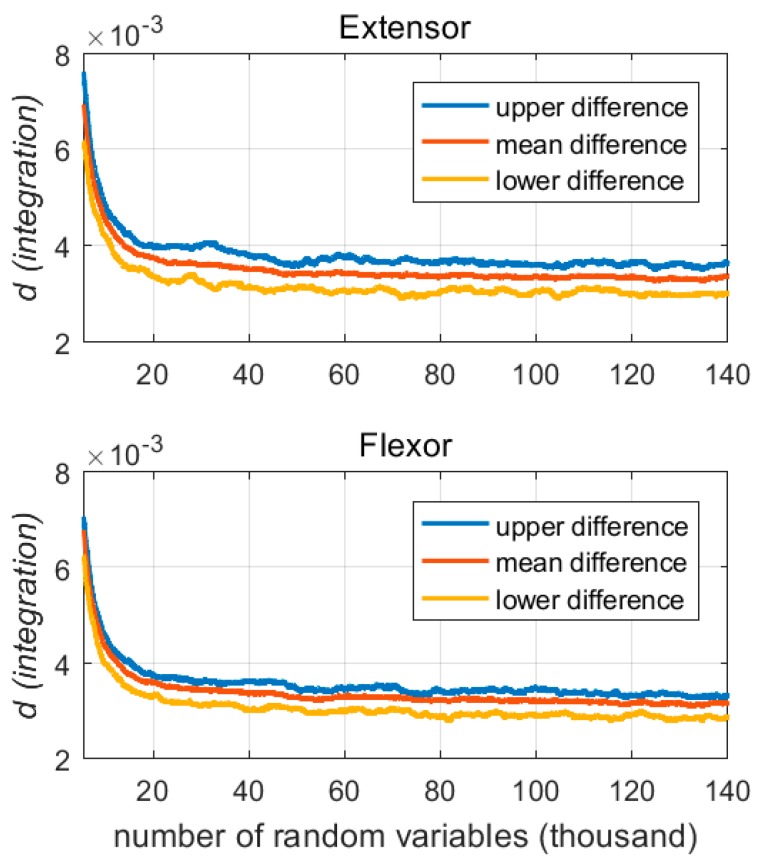
The results of the convergence experiment for determining the number of random variables.

In this paper, the random variable was distributed evenly in a specific range (0–100) as a basic step to realize the Monte Carlo simulation. In each iteration of the simulation, each model parameter used a given number of random variables to obtain the expected sensitivity value. The given number of random variables was obtained by a finite difference-based convergence experiment for all participants in Group A [27]. This experiment showed a relationship between the number of random variables and the expected sensitivity estimator, as shown in Figure 4. This experiment was performed at a specific variable number range, *r*, that was between 100 to 140,000, with an interval of 100. By using this range, *r*, the sensitivity of each wavelet scale was obtained from the Monte Carlo simulation at each given variable quantity and these sensitivities were calculated by first order differential calculation.

(4) $$d_{i}\left(p\right)=\left|y_{i+1}\left(p\right)-y_{i}\left(p\right)\right|$$

(5) $$i=\frac{r}{100}$$

where *p* = 1, 2,…, 10, is the ordinal number of the wavelet scale, *r* = 100, 200..., 140,000, and *y_i_*(*p*) is the sensitivity value of the wavelet scale of *p* with a specific random variable number (*r* = *i* × 100). Then, the sum, *d_i_*(*integration*), of the sensitivity differences under a variable number for Group A can be calculated as follows:

(6) $$d_{i}\left(integration\right)=\overset{10}{\underset{p=1}{\sum }}d_{i}\left(p\right)$$

Figure 4 depicts the influence of the number of random variables on the sensitivity distribution. The mean difference (red line) decreased as the number of random variables increased, and tended to stabilize from 60,000. The upper difference (blue line) and the lower bound difference (yellow line) became stable when the number of random parameters exceeded 100,000. Therefore, this paper used 100,000 random parameters, which were uniformly distributed between 0–100, to perform the Monte Carlo simulation for analyzing the sensitivity of each wavelet scale to handgrip force.

#### 2.3.3. Sequence Combination Analysis (SCA)

**Figure 5:**
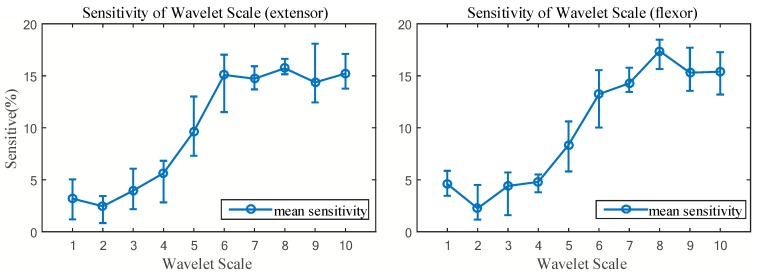
Sensitivity distribution of the ten wavelet scales to handgrip force.

**Figure 6:**
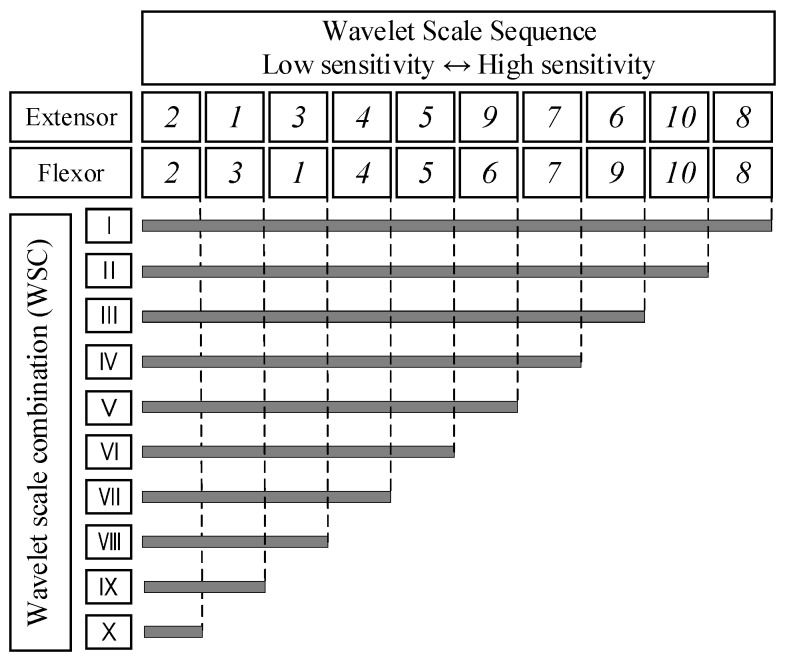
Sequence combination of wavelet scales based on the sensitivity value.

Based on the Monte Carlo simulation, we obtained the nonlinear degree of each scale-handgrip force correlation through the force-varying analysis tasks. To choose the suitable WSC, we designed a set of scale permutations and combination rules based on the mean value of sensitivity of each scale. The mean sensitivity value was calculated from all participants in Group A. The distribution and mean sensitivity value of each scale is shown in Figure 5. Through frequency band analysis, Soo et al. (2010) observed that wavelet scales 2 and 3 have large effects on fatigue resistance during muscle isometric contractions [6]. By using the Monte Carlo sensitivity analysis, it was shown that these two scales have lower sensitivity to handgrip force than other scales. Therefore, this paper determined a specified sequence and combination rule of the wavelet scale. The rule is that the scale with low sensitivity is preferred for retention and the scale with high sensitivity is eliminated, in turn. By considering the wavelet scales described in Section 2.3.1, we can obtain ten WSCs (combination serial numbers I, II, III, IV, V, VI, VII, VIII, IX, X) based on the proposed sequence combination analysis. The details of the WSCs are illustrated in Figure 6. The coefficient tern structure and parameters of the original nonlinear SEMG-handgrip force relationship were adjusted to match the different WSCs. This paper uses RMSE as the evaluation index to determine which WSC has the appropriate estimation accuracy for handgrip force. 

### 2.4. Performance Comparison

#### 2.4.1. Former Method

In this paper, two former methods were utilized to be compared with the proposed method. Formerly, force estimation was realized by calculating the amplitude of the SEMG signal, for example, using RMS and the pre-defined SEMG-force relationship. Former method A builds a linear SEMG-force relationship by using the normalized SEMG signal, which is bandpass-filtered between 15 and 450 Hz [5]. Former method B builds a SEMG-force model based on the frequency-band technique, which determines a suitable WSC (wavelet scales 2 and 3) to estimate the handgrip force from SEMG signals during an isometric muscle contraction task [6].

#### 2.4.2. Performance Index

To obtain the estimated error, a comparison between the estimated and actual handgrip force was conducted using the RMSE:

(7) $$\mathrm{RMSE}=\sqrt{\frac{1}{T}\overset{T}{\underset{t=1}{\sum }}{\left(F{\left(actual\right)}_{t}-F{\left(estimation\right)}_{t}\right)}^{2}}$$

where *F(actual)* is the sample data of the observed griping force from the grip dynamometer and *F(estimation)* is the predicted force from SEMG. The sampling number of handgrip force is  1≤t≤T. A value of RMSE closer to zero indicates that the model has a smaller random error component.

## 3. Results and Discussion

### 3.1. Nonlinear Correlation between Wavelet Scale and Handgrip Force 

Figure 5 describes the distribution between the wavelet scale and its sensitivity to handgrip force for Group A. These sensitivity values can be used to evaluate the degree of nonlinear correlation between the wavelet and handgrip force. According to the observation of the sensitivity distribution of the two muscles, scales 1, 2, 3, 4 have lower degree nonlinear correlations with handgrip force, the degree of nonlinear correlation between scales 6, 7, 8, 9, 10 and handgrip force are generally higher, and scale 5 is in the middle sensitivity position. According to Soo et al. (2010), the four wavelet scales with a low degree of nonlinear correlation to handgrip force are in the 181.82–727.27 Hz central frequency range, the five scales with a higher degree of nonlinear correlation to handgrip force are between 72.72 Hz and 121.21 Hz of central frequency and the center frequency of scale 5 is in the middle at 145.45 Hz [6]. Wakeling et al. (2002) reported that the frequency ranges for recruitment of slow and fast twitch muscle fibers are approximately 143 and 397 Hz of SEMG, respectively [33]. Thus, it can be approximated that slow fiber signal actions at scale 5, and fast fibers, are located at low sensitivity scales. Begg et al. (2007) reported that the SEMG of low frequency signals contain motion artifacts [34], which may result in high sensitivity. Many researchers reported that the power spectrum of SEMG shifts to a lower frequency range when muscle fatigue develops [11,18,19]. According to the center frequency of the wavelet scale, the wavelet scales 6–10 belong to the low frequency range of the SEMG signal. This is the reason why the wavelet scales of low frequency signals (i.e., scale 6–10 in Figure 5) have a high degree of sensitivity.

From the results shown in Figure 5, we can observe that each scale has a different sensitivity distribution. To simplify the distribution, we calculated the mean sensitivity of each scale to express the relationship between the ten wavelet scales and their sensitivity to handgrip force. Then, wavelet scales were arranged, in ascending order, in regard to mean sensitivity value, as shown in Figure 6.

### 3.2. Selection of Wavelet Scale Combination

Figure 6 describes a layout of the SCA. The layout shows the sequence and combination of the ten wavelet scales. For instance, the WSC II covered all scales, except scale 8, and the WSC X only had scale 2. The performances of force estimation under the ten WSCs were evaluated by RMSE during the force-varying validation tasks. The RMSE was calculated based on the difference between the handgrip force estimated from SEMG and the handgrip force measured from the grip dynamometer.

**Figure 7:**
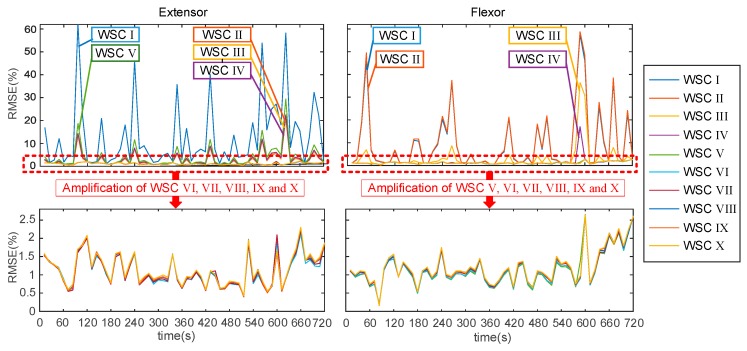
Varying RMSE process of each wavelet scale combination (WSC) in the force-varying validation task.

**Figure 8:**
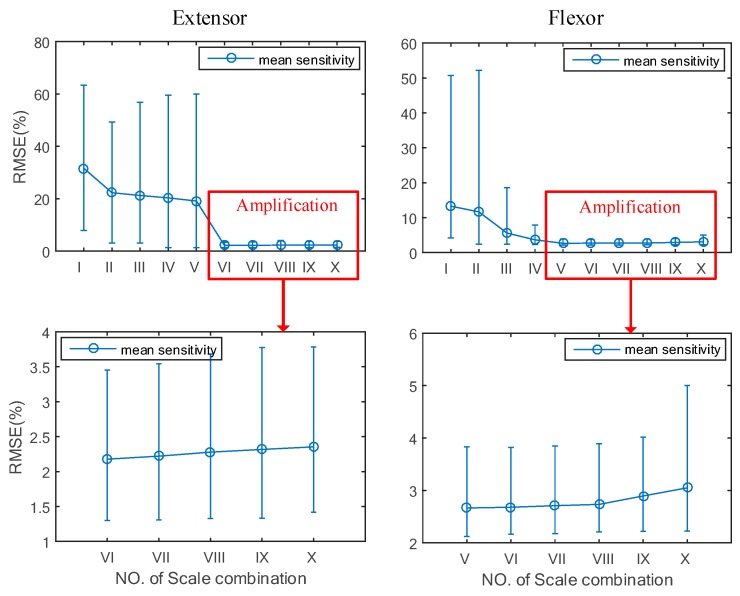
Comparison of the ten WSCs.

The performance evaluation consisted of a varying RMSE process of the ten WSCs during the force-varying validation task. Figure 7 illustrates that the performance evaluation of the ten WSCs is a time-varying RMSE process. Each WSC has a different error curve during the force-varying validation task. Figure 7 shows that WSC I, II, III, IV, and V obtained from the extensor muscles have unsatisfactory estimated accuracies, and WSC VI to X have similar estimation accuracies in the amplificatory graph. In comparison to the five WSCs (I, II, III, IV, and V), the removal of highly sensitive wavelet scales can help to improve the estimation accuracy of handgrip force over a long duration griping motion. WSC I, II, III and IV obtained from the flexor muscle have unsatisfactory accuracies of estimated force, and WSC V to X have similar estimated accuracies. To choose the suitable WSC, a statistical method was used to evaluate the differences between estimated accuracy for ten WSCs, as shown in Figure 8.

Figure 8 shows the handgrip force estimation error that was derived from Group A under different WSCs. The WSCs of I, II, III, IV, and V in the extensor muscle have larger predicted errors of handgrip force than the others. However, after amplification of local errors, it was found that the minimum force estimation error occurred in WSC VI. The suitable WSC selection for the flexor muscle is similar to that for the extensor muscle. The WSC V of the flexor muscle is considered to have the minimum RMSE range and mean in estimation of the long duration handgrip force from SEMG.

### 3.3. Performance Comparison

**Figure 9:**
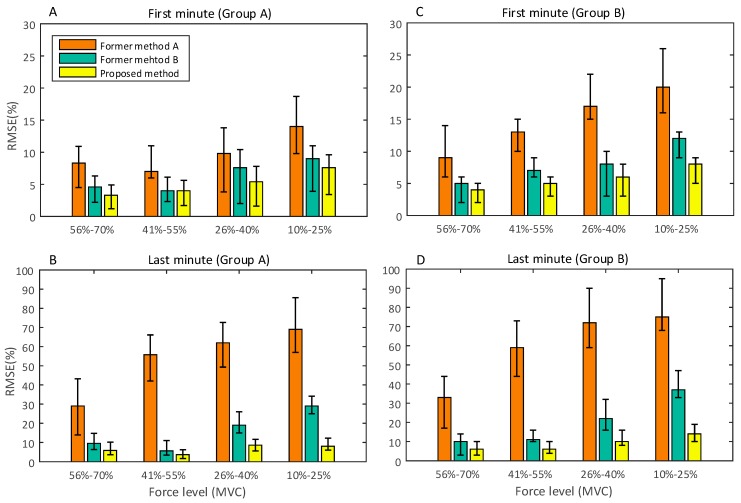
Results of the force-varying validation tasks.

**Figure 10:**
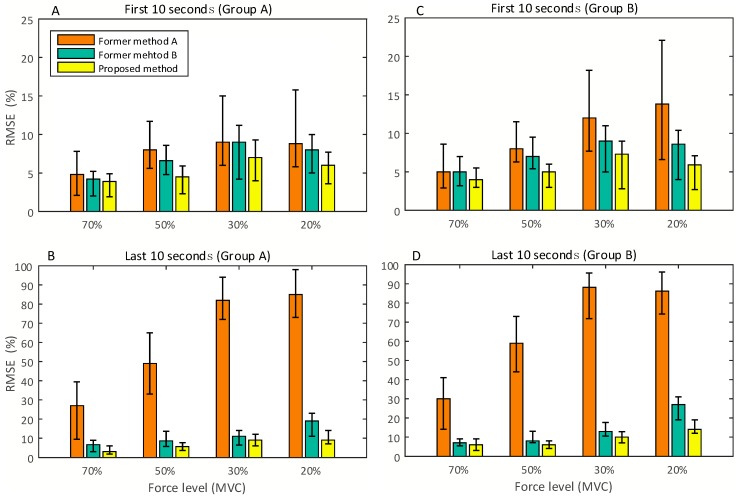
Results of the static validation tasks.

Figure 9 and Figure 10 show the comparisons between RMSE of handgrip force, estimated by the proposed method and former methods, in both force-varying and static validation tasks. Figure 9A,C present the RMSE obtained by the participants in Groups A and B within the first minute of the force-varying validation task. In the first minute of the task, the SEMG is affected by a low degree of physiological effect of fatigue and motion artifact. Figure 9B,D shows the performance comparisons in the last minute of the validation task. In the last minute of task, the complex noise (electrophysiological effect of fatigue, motion artifact, and so on) seriously affects SEMG amplitude and frequency, and reduces the correlation between SEMG and handgrip force. This is the reason why the RMSE in the first minute is smaller than that in the last minute. Figure 10 shows the RMSE derived by the proposed method and former methods during the static task. Since the static task is more likely to produce fatigue than the force-varying task, we compared the estimated accuracy of these three methods in the first 10 s and the last 10 s of the isometric contraction task, at four force levels. The results show that the RMSE in the first 10 s is smaller than that in the last 10 s.

In the force varying validation task, the experimental results (Figure 9) show that the proposed method has a better performance than the others in terms of force estimation, especially in the force range of 10–25%. This is because the proposed method can obtain a better pre-defined SEMG-handgrip force relationship than the former methods. In contrast with the high degree of force (greater than 41% MVC), the performances of these three methods are slightly different. Because high force muscle contraction requires a large number of motor units to maintain the particular level of force, the high force muscle contraction is different from the low force contraction. Based on the principle of motor unit recruitment [1,9], the three methods can obtain a similar pre-defined SEMG-handgrip force relationship at a high force level. Since former method A contains all wavelet scales, except for scale 1, its pre-defined SEMG-handgrip force relationship is based on the scales with considerable noise (motion artifacts and random noise); thus, the performance of this method is weak compared to the other methods. The similar results can be obtained in the static validation task, as shown in Figure 10.

The proposed method has a significant performance improvement for fatigued muscle contractions with long durations (see Figure 9B,D and Figure 10B,D). The proposed method reduces the force-varying validation RMSEs by 27%, 53%, 54%, and 61%, compared to former method A, in the handgrip force ranges of 56–70%, 41–55%, 26–40% and 10–25% of MVC, respectively. The RMSE of the proposed method is reduced by 4%, 2%, 11% and 21%, respectively, in these four ranges of force, compared to former method B. In the static validation tasks, the proposed method can significantly improve the performance of force estimation at the low force level. A large amount of noise caused by the electrophysiological effect of fatigue and motion artifacts affects the amplitude and frequency of SEMG, which causes the pre-defined SEMG-handgrip force relationship built by former methods to no longer be accurate.

Compared to the first minute, the estimated accuracy of former method B in the last minute decreased by an average of 11% in the force range of 10–40% of MVC. From the perspective of sensitivity of each scale to handgrip force, this is because the wavelet scales 2 and 3 have lower sensitivities to gripping force prediction than the other scales and thus, cannot characterize flexibles change during force-varying contractions for low forces. The signal noise induced by the intramuscular electrophysiological effect and motion artifacts has consequences for the correlation between the two scales and handgrip force and further reduces the estimated performance of former method B. Previous studies have shown that low strength muscle contractions are mainly controlled by slow-twitch muscle fibers (approximately 143 Hz of SEMG), and high strength muscle contractions are mainly controlled by fast-twitch muscle fibers (approximately 397 Hz) [33,35]. In former method B, the SEMG signal of 143 Hz is missing due to the limitation of scale pre-layout in frequency band analysis technology [6]. This is the reason why the performance of former method B is worse than our proposed method.

In contrast to the former methods, the proposed method can effectively inhibit the influence of complex electrophysiological noises on the predicted accuracy of the handgrip force from SEMG during long duration fatiguing muscle contractions. However, the prediction errors for low-level forces are greater than those for high level forces. The reason for this is that the intramuscular electrophysiological effect and motion artifacts can reduce the degree of correlation between different levels of force and SEMG to varying degrees. During the long duration muscle contractions, all participants overcame fatigue to produce a specified handgrip force with extra shaking of the forearm. Motion artifact noise may be caused by the shaking of these forearm muscles. When the fatigue reduces the force generated from the muscle, the rotation and substitution between fatigued motor units and fresh motor units is performed to generate and maintain the requisite force level. In this situation, the complex electrophysiology within muscles affects the changes in the SEMG signal. Thus, the intramuscular electrophysiological effect and motion artifacts ultimately change the amplitude and frequency of the SEMG signal. 

## 4. Conclusions

In this paper, we proposed a nonlinear correlation-based method to select appropriate wavelet scales to accurately estimate handgrip force from SEMG during a force-varying muscle contraction of long duration. The sensitivity analysis of the Monte Carlo simulation was used to explore the nonlinear correlation between ten wavelet scales and handgrip force. A nonlinear, correlation-based SCA method was proposed to determine the suitable WSC. The results derived via SCA indicated that the suitable scales for the extensor muscles is WSC VI, consisting of scales 1 to 5, and WSC V including scales 1 to 6 is suitable for flexor muscles. The appropriate WSC, obtained by the proposed method, was compared to two former methods through a set of static and force-varying contraction tasks. The experimental results showed that the proposed method has a better estimation performance than the former methods, especially for low level contractions (0–30% maximal voluntary contraction).

The proposed method enhances the performance of grip strength estimation from SEMG. However, the actual working muscles not only provide force output but also drive limb posture changes. Further research using the proposed nonlinear correlation-based scale selection method is needed to explore the correlation between wavelet scales of SEMG and muscle loads or limb posture in various work tasks, such as human–robot interactions, and gait analysis based on SEMG of lower limb muscles. An accurate force estimation based only on electromyography data can be used for gait analysis instead of using inverse dynamics or similar methods [36,37]. In addition, the participants in this study were limited in age, health, and sex ratio (i.e., 16 healthy male students and four healthy female students); thus, further research should take into account the gender differences in the results of the proposed method.
